# Determinants of Chronic Biological Stress, Measured as Hair Cortisol Concentration, in a General Population of Adolescents: From Individual and Household Characteristics to Neighborhood Urbanicity

**DOI:** 10.3389/fpubh.2021.669022

**Published:** 2021-11-23

**Authors:** Veerle J. Verheyen, Sylvie Remy, Eva Govarts, Ann Colles, Gudrun Koppen, Laura Rodriguez Martin, Flemming Nielsen, Liesbeth Bruckers, Esmée M. Bijnens, Stijn Vos, Bert Morrens, Dries Coertjens, Ilse Loots, Annelies De Decker, Carmen Franken, Elly Den Hond, Vera Nelen, Stefaan De Henauw, Adrian Covaci, Nicolas Van Larebeke, Caroline Teughels, Tim S. Nawrot, Greet Schoeters

**Affiliations:** ^1^VITO Health, Flemish Institute for Technological Research (VITO), Antwerp, Belgium; ^2^Department of Biomedical Sciences, University of Antwerp, Antwerp, Belgium; ^3^Institute of Public Health, Department of Environmental Medicine, University of Southern Denmark, Odense, Denmark; ^4^I-BioStat, Data Science Institute, Hasselt University, Hasselt, Belgium; ^5^Centre for Environmental Sciences, Hasselt University, Hasselt, Belgium; ^6^Department of Sociology, Faculty of Social Sciences, University of Antwerp, Antwerp, Belgium; ^7^Provincial Institute of Hygiene, Provincial Research Centre for Environment and Health, Antwerp, Belgium; ^8^Department of Public Health and Primary Care, Faculty of Medicine and Health Sciences, Ghent University, Ghent, Belgium; ^9^Toxicological Centre, University of Antwerp, Antwerp, Belgium; ^10^Analytical, Environmental and Geo-Chemistry, Vrije Universiteit Brussel, Brussels, Belgium; ^11^Department of Radiotherapy and Experimental Cancerology, Ghent University, Ghent, Belgium; ^12^Flemish Planning Bureau for the Environment and Spatial Development, Brussels, Belgium

**Keywords:** adolescents, chronic biological stress, determinants, hair cortisol concentration, Flemish environment and health study (FLEHS), neighborhood urbanicity

## Abstract

Chronic biological stress may adversely affect adolescents' physical and mental health, but insight in the personal and environmental factors that determine chronic stress is limited. We measured 3-month cumulative hair cortisol concentration (HCC) in 419 adolescents, participating in the Flemish Environment and Health Study. Adolescents' health and lifestyle characteristics, household and neighborhood socio-economic status as well as neighborhood urbanicity were assessed as potential determinants of HCC, using multiple linear regression models. We additionally explored heterogeneity of our results by sex. HCC were significantly higher in boys from densely populated neighborhoods, the association was not significant in girls. Accordingly, boys living outside cities had significantly lower HCC than boys, living in cities. HCC was significantly lower in adolescents with an optimal vitality, a measure of a positive mental health status. In adolescent girls, menarcheal status (pre-/postmenarche) was a significant determinant of HCC. Our findings are the first to suggest that residential urbanicity may have an impact on chronic biological stress in a general population of adolescent boys.

## Introduction

Adolescence is characterized by many physical, emotional, social and cognitive challenges (1). Chronic biological stress during this vulnerable developmental period may have long-lasting implications for physical and mental health (2, 3). The hypothalamic–pituitary–adrenal (HPA) axis, with the hormone cortisol as its main effector, is a crucial biological stress response system (4). Acute and transient activation of the HPA axis facilitates effective coping with stressors (5). However, chronic HPA axis activation may lead to long-term physiological alterations and has been associated with respiratory and cardiovascular diseases, type 2 diabetes, cognitive disorders and depression (4, 6). A good insight in the factors that determine chronic HPA axis activation and cortisol secretion in adolescents is important, however, these factors are not yet fully established (7).

Traditional assessment of HPA axis function through measurement of cortisol levels in saliva, blood or urine, reflects cortisol concentrations over minutes to 24-h prior to sampling. Obtaining valid information on long-term cortisol secretion is difficult using these matrices (8). In the past decade, the cortisol concentration in scalp hair has emerged as a biomarker of long-term HPA-axis activity (4). Sampling is easily conducted and non-invasive, a single hair sample retrospectively captures several months of cortisol secretion (9). The number of studies using hair cortisol concentration (HCC) as a biomarker of chronic biological stress has substantially increased in the past years. However, literature on determinants of HCC in healthy adolescents is still limited. Most existing studies were conducted in the context of physical and mental diseases or in socioeconomically disadvantaged subgroups and included both children and adolescents (10). A systematic review of literature (7), identified sex, age, anthropometry and household socioeconomic status (SES) as determinants of HCC in children and adolescents. Recent studies, including only adolescents, did not find significant associations of HCC with sex; studies on HCC in relation to anthropometry yielded varying results (11–14). Gray et al. (7) identified associations of HCC with perinatal characteristics, atopic illness and mental health as important remaining knowledge gaps. The perinatal period may influence HPA functioning and disease risk later in life (15). Atopic disease and the use of corticosteroid medication may also affect HPA axis functioning (16). No significant associations of HCC with adolescents' mental health measures, including perceived stress or anxiety, have yet been reported (7, 10, 17). Interestingly, a small study among adolescents (12–18 years, *n* = 27) did not find a significant association of HCC with perceived stress, however, adolescents' optimism was significantly associated with lower HCC (18).

The HCC of adolescents may also be related to their residential environment. An urban, densely populated, residential environment has been hypothesized to act as a chronic stressor, regardless of a person's individual socioeconomic position (19, 20). Potential pathways are the social context of an urban neighborhood and a higher exposure to air pollution, noise and heat (21, 22). In an exploratory study among children (median age 10 years, *n* = 92), neighborhood urbanicity was not related to HCC (20). However, neighborhood socio-environmental influences may exert stronger effects on adolescents, compared to children (19).

The objective of this study was to gain a better insight in sociodemographic, health and lifestyle factors as well as neighborhood characteristics that contribute to chronic stress in adolescents. The selection of potential determinants of chronic biological stress was based on existing literature. To the best of our knowledge, this study is the first to explore HCC in relation to neighborhood urbanicity in adolescents. During adolescence the responsivity of the HPA axis may be sex-dependent. We therefore additionally explored effect modification of associations between HCC and potential determinants by sex (23, 24). Our study may support formal and informal initiatives to promote adolescents' health and wellbeing through chronic biological stress prevention and reduction.

## Materials and Methods

### Study Population and Design

This study was embedded in a human biomonitoring program, i.e., the Flemish Environment and Health Study (FLEHS), initiated by the Flemish government in 2002. The goal of FLEHS is to investigate the relationship between a broad range of environmental exposures and health effects and to support environmental health policy by identifying priorities for further action (25). This study was conducted within the fourth cycle of the FLEHS (FLEHS-4, 2016–2020), including adolescents from the general population of Flanders. One of the main objectives of FLEHS-4 was to investigate associations of residential environmental surroundings with adolescents' health. The study protocol was approved in June 2017 by the Antwerp University Hospital Ethical committee (registration number B300201732753). Between September 2017 and June 2018, a representative sample of 428 Flemish 14–15 year old adolescents was recruited and examined in schools by trained nurses. Parents and adolescents filled out extensive questionnaires that provided detailed information on health, lifestyle and household socio-economic status. A stratified clustered multi-stage sampling strategy was applied to enroll equal numbers of participant across both sexes, to represent all educational levels, to geographically represent all Flemish provinces and different degrees of urbanicity. The inclusion criteria of the FLEHS-4 study were: informed consent signed by the adolescent and a parent, having lived in Flanders for at least 5 years, adolescent and parents mastered enough Dutch to fill out questionnaires. Exclusion criteria were: data of more than one questionnaire missing, blood and urine sample missing, being held back in school for more than 1 year, attending a boarding school. One adolescent was excluded because of pregnancy. Of the 428 FLEHS-4 participants, eight could not participate in this part of the study because their hair was too short (<3 cm). One participant was excluded for following a growth hormone therapy. Thus, the final sample of this study included 419 adolescents.

### Hair Cortisol Concentrations

A strand of hair of at least 3 cm was cut close to the scalp from the posterior vertex of the adolescents' head. Hair samples were stored in the dark at room temperature in paper envelopes. Analysis was performed within 18 months after collection of the first samples. HCC was determined from the scalp-near 3 cm hair segment. Human hair on the head grows at a rate of approximately 1 cm per month, HCC of 3 cm hair strands retrospectively reflect cortisol levels for the period of 3 months (26). Samples were analyzed at the Institute of Public Health, Department of Environmental Medicine of the University of Southern Denmark (SDU), using liquid chromatography combined with tandem mass spectrometry (LC-MS/MS) as described previously by (27). Briefly, hair samples were washed with methanol and dried at room temperature. The 3 cm hair strand was cut into 2–3 mm segments that weighed 20–30 mg. Aliquots of 100 μL 20 ng/mL isotope labeled cortisol-D4 were added as internal standard, together with 0.9 mL methanol. Samples were incubated in the dark at 25°C, while whirl mixed at 2,000 revolutions per minute for 5 days and subsequently centrifuged at 3,000 g for 5 min. Twenty microlitre of the supernatant was injected onto a High-Performance Liquid Chromatography (HPLC) column. HPLC was performed using an Accella 1,250 pump (Thermo Scientific, San Jose, CA) and a PAL autosampler (CTC analytics, Zwingen, Switzerland). The analytical column was a Kinete × C18 column, 100 × 4.6 mm (2.6 μm) equipped with a 2 × 4 mm C18 SecurityGuard column (Phenomenex, Torrance, CA). Isocratic elution was performed with a mobile phase system consisting of methanol and 0.1 M formic acid (80:20) at a flow rate of 400 μL/min for 6 min. After the peaks were eluted, a wash procedure was performed before the next samples was injected onto the column. The triple quadrupole mass spectrometer was a TSQ Vantage (Thermo Scientific, San Jose, CA). The calibration curve and calculation of the sample concentration were based on the area ratio of the analyte/isotope labeled internal standard. The calibration curve ranged from 0.02 up to 200 ng/mL, which corresponds to a range of 1–1000 pg/mg hair when 20 mg of hair is utilized. Quality control samples were included in the analysis with low, medium and high concentration levels (7.1, 35.9 and 91.7 pg/mg, respectively). The limit of quantification (LOQ) for cortisol was 0.3 pg/mg hair. The intra-day repeatability coefficient of variation (CV) of the assay was 8.7% and the inter-day reproducibility CV was 9.5%.

### Determinants of Hair Cortisol Concentrations

#### Sociodemographic Variables

The country of birth of the adolescent and his or her parents was assessed as Belgium, European Union, or outside the European Union. Household socioeconomic status (SES) was evaluated, based on education and income variables. Therefore, the highest household educational attainment was assessed according to the Belgian education system as primary (no educational attainment, primary school, lower secondary school), secondary (higher secondary school) and tertiary (higher education attainment). Monthly equivalent income was calculated as the total monthly household income standardized to the number of household members, a value of one is assigned to the household head, of 0.5 to each additional adult member and of 0.3 to each child under 18 year (28). Parents also reported their perceived income adequacy, a subjective SES measure, ranging from difficult to very easy to make ends meet.

We geocoded participants' homes addresses to assess neighborhood urbanicity and neighborhood SES. We classified urbanicity of the participants' residential neighborhood, using the Eurostat definition of urbanicity that is based on a combination of geographical contiguity and population density, applied to 1 km^2^ population grid cells (29). Eurostat defines neighborhoods as (1) cities: densely populated areas where 50% or more of the population lives in urban Centers with a population density of at least 1,500 inhabitants per km^2^ and at least 50,000 inhabitants, (2) towns/suburbs: intermediate density areas where 50% or more of the population lives in urban clusters with a population density of at least 300 inhabitants per km^2^ and a minimum population of 5,000 inhabitants, (3) rural areas: thinly populated areas outside of city Centers and urban clusters.

In accordance with previous FLEHS studies, we assessed population density at the municipality level dichotomously as low residential population density (≤ 600 inhabitants/km^2^) and high residential population density (>600 inhabitants/km^2^) (30). We assessed neighborhood socioeconomic status (SES) using the Area Deprivation Index (ADI), which is calculated at a sub-municipality level in Flanders on a yearly basis (31). The area deprivation index of 2018 considers all children born in year 2018, 2017 and 2016 that live in deprived households in a given neighborhood in Flanders, divided by the total number of children born in this neighborhood during the same period. The ADI is documented by the Child and Family Government Agency (www.kindengezin.be). Selection criteria are the family's monthly income, the parents' educational attainment and employment situation, development of the children, housing and health. If a family fulfills at least three criteria, it is considered to be deprived.

#### Lifestyle, Physical and Mental Health Variables

Information on adolescents' lifestyle included smoking, frequency of alcohol use and sports (physical activity that causes sweating and/or heavy breathing). We gathered information on their hair washing frequency and hair treatment (dying, bleaching).

During sampling at school, waist circumference, body length and weight were measured by study nurses with standardized equipment and according to standardized protocols. From this information, the body mass index (BMI) was calculated as body weight in kg/(body length in m)^2^. Boys and girls were classified as underweight, normal weight, overweight or obese according to the sex- and age-specific 2004 Belgian growth curves (32). The parent questionnaire provided information on perinatal factors, including maternal smoking during pregnancy, low birth weight (<2500 g) and preterm birth (born before the 37^th^ pregnancy weeks). Atopic disease (doctor-diagnosed asthma, atopic dermatitis, allergic rhinitis in the past year) was evaluated, based on questions from the International Study of Asthma and Allergy in Childhood (ISAAC) questionnaire (33). The use of corticosteroid medication in the 14 days prior to sampling was reported and information on infections in the past year was also provided. Girls provided information on their menarcheal status and use of contraceptives.

Mental health of adolescents was assessed by the following indicators:

- The Strengths and Difficulties Questionnaire (SDQ), a validated screening method that covers a broad range of mental health symptoms (34, 35). The 25-item versions of the SDQ contains four scales, each of five items, focusing on difficulties in emotional functioning, conduct, hyperactivity and interaction with peers in the past 6 months (35). The five items can be scored 0, 1, or 2, and the total score for each scale can therefore range from 0 to 10. These four scales together form the total difficulties scale (range 0–40). Additionally, the SDQ contains one 5-item scale focusing on prosocial behavior. A high score on the total difficulties scales (20–40) represents an abnormal degree of difficulties; a high score on the prosocial scale (6–10) represents normal prosocial behavior.
- The Vitality scale for positive mental health from the Short Form-36 (SF-36) Health Survey questionnaire assesses vitality based on four items: lust for life, energy level, level of exhaustion and tiredness in the past 4 weeks, with scores between 0 and 100 (best score) (36). To reflect optimal vitality, a cut-off value of 77.4 was applied to the continuous vitality score, being the study mean vitality score plus one standard deviation, in accordance to the BHIS, the Belgian Health Interview Surveys (37). Optimal vitality was evaluated dichotomously for each participant as a value above the cut-off value.
- Perceived stress in the past 3 months was assessed based on the question “Did you experience a lot of stress in the past 3 months?” and was scored as low (never, seldom), medium (sometimes), high (often, always).
- Self-rated general happiness was evaluated, based on the question “All things considered, would you say that you are happy?” and was scored as low (not happy at all, rather not happy) or high (rather happy, very happy).

#### Meteorological Data

Data on local ambient temperature and UV-radiation was provided by the Belgian Royal Meteorological Institute (KMI). Temperature was measured in 15 weather stations in Flanders, we used data from the weather station closest to the adolescents' home to calculate average temperature and UV-radiation over the 3-month period prior to sampling. Based on the sampling date, the season of sampling was determined.

### Statistical Analysis

Statistical analysis was performed using SPSS Statistics (version 26; IBM, Armonk, NY, USA) and R version 3.5.0 (R Foundation for Statistical Computing, Vienna, Austria). HCC concentrations (pg/mg hair) were not normally distributed and were therefore transformed by the natural logarithm prior to statistical analysis. For two HCC values below the LOQ, values were imputed using a truncated lognormal distribution. First a truncated lognormal distribution was fitted for the observed values (the values above the LOQ). This resulted in an estimate of the mean and standard deviation of the lognormal distribution of all values (below and above the LOQ). For values < LOQ, random values were imputed, taken between 0 and the limit from the lognormal distribution with the estimated mean and SD. Outliers (above the mean + 3 standard deviations) were not excluded from our main analyses, since we found no analytical or biological reason for exclusion. HCC are described as the geometric mean concentrations with 95% confidence interval (95% CI), together with the median HCC, 25^th^, 75^th^ and 90^th^ percentile. HCC of boys and girls were compared using an independent samples *t*-test.

We first examined associations between HCC as a dependent variable and each determinant as an independent variable in linear regression models, adjusted for age and sex. A *p* ≤ 0.05 was used as cut-off for statistical significance. Effect modification by sex was assessed by adding the interaction term of each determinant and sex into the regression model and evaluating the *p*-value of the interaction term. Only significant interactions (*p* ≤ 0.10) were reported. The use of contraceptives and menarcheal status were evaluated as potential determinants of HCC in girls-only models.

Next, multiple linear regression models were built, including significant determinants of HCC (*p* ≤ 0.05), additionally adjusted for variables that were associated with HCC at a significance level of ≤ 0.20 in the former analysis. Age and sex remained in the models as fixed covariates regardless of their significance. We examined associations between the independent variables by Spearman rank correlation (ρ). Collinearity between independent variables in the models was checked by evaluating the variance inflation factor (VIF <3) (38). The R-square of the model is presented as the percentage of variation in HCC, explained by the independent variables in the final model.

In a sensitivity analysis, all associations were evaluated after winsorizing HCC values that were identified as outliers (39). In line with previous cortisol research (9, 40–42), HCC values of more than 3 standard deviations (SD) above the mean were set at 3 SD above the mean before transformation to the natural logarithm, to reduce their impact on data analysis.

Given the large selection of potential determinants that was included in this exploratory analysis, we accounted for multiple testing. We verified our results after controlling for a false discovery rate (FDR) at *q* = 0.10 using the Benjamini and Hochberg method (43). We included the *p*-values of each association between an independent variable and HCC after adjustment for age and sex in the Benjamini–Hochberg procedure. For variables that were significantly modified by sex, we included *p*-values of each sex separately.

## Results

### Study Population Characteristics

Sociodemographic, lifestyle and health characteristics of the study population are described in Table 1. Our study population consisted of 419 adolescents. Slightly more girls (*n* = 228, 54.4%) participated in this study compared to boys (*n* = 191, 45.6%), but equal distribution between the sexes is approached. The mean (SD) age of the study population was 14.8 (± 0.5) years for both sexes. For 80.4% of the adolescents, Belgium was their country of birth; this proportion reflects the general Flemish population (79.5% born in Belgium) (44). Household educational attainment was high in this study: 59.4% of the households had a parent that achieved a tertiary educational level compared to 41% in Flanders (45). This tendency was also observed in previous FLEHS studies and is due to better response rates in highly educated households (46, 47). The average ADI of 12% in our study population is slightly lower than the Flemish ADI of 14.5% (48). Almost one out of three households (28.4%) found it difficult to make ends meet with their income. Residential population density was high for 36% of participants whereas 48.9% of the Flemish population lives in densely populated municipalities (44). A minority of participants lived in cities (13.6%) and rural areas (14.1%), the majority lived in towns and suburbs (72.3%). On the national scale, a higher share of 29.5% of the population lives in cities, 55% of Belgians live in towns and suburbs, and comparable to our study population, 15.5% live in rural areas (49). BMI was normal for 77% of the participating boys and 68% of the girls, this is comparable to the fraction of Flemish adolescents (10–17 years) with a normal BMI (75.2 and 69%, respectively) (44). Vitality was optimal for 13.4% of participants, a result in line with the 14% of the Belgian population (50). More than one third of participants (33.2%) reported to perceive a lot of stress.

**Table 1:** Study population characteristics.

**Characteristics**	**All (*****n*** **=** **419)**		**Boys (*****n*** **=** **191)**		**Girls (*****n*** **=** **228)**	
	***n***	**%**	***n***	**%**	***n***	**%**
**Sociodemographic variables**						
Age (years)						
[13.5–14.5] years	113	27	48	25.1	65	28.5
[14.5–15.5] years	273	65.2	127	66.5	146	64
>15.5 years	33	7.9	16	8.4	17	7.5
Country of birth						
Belgium	337	80.4	158	82.7	179	78.5
EU	35	8.4	15	7.9	20	8.8
Outside EU	46	11	18	9.4	28	12.3
Missing	1	0.2	0	0	1	0.4
Household educational attainment						
Primary	24	5.7	6	3.1	18	7.9
Secondary	139	33.2	53	27.7	86	37.7
Tertiary	249	59.4	131	68.6	118	51.8
Missing	7	1.7	1	0.5	6	2.6
Equivalent income (monthly)						
0–1250 Euro	89	21.2	40	20.9	49	21.5
1251–1600 Euro	73	17.4	35	18.3	38	16.7
1601–2000 Euro	71	16.9	31	16.2	40	17.5
>2000 Euro	118	28.2	57	29.8	61	26.8
Missing	68	16.2	28	14.7	40	17.5
Perceived income adequacy						
Difficult	119	28.4	56	29.3	63	27.6
Rather easy	133	31.7	62	32.5	71	31.1
Easy to very easy	160	38.2	70	36.6	90	39.5
Missing	7	1.7	3	1.6	4	1.8
Area deprivation index						
0–5.3%	103	24.6	42	22	61	26.8
5.4–9.3%	105	25.1	42	22	63	27.6
9.4–15.5%	103	24.6	48	25.1	55	24.1
>15.5%	107	25.5	58	30.4	49	25.1
Missing	1	0.2	1	0.5	0	0
Urbanicity (Eurostat)						
Rural areas	59	14.1	17	8.9	42	18.4
Towns/suburbs	303	72.3	154	80.6	149	65.4
Cities	57	13.6	20	10.5	37	16.2
Population density						
<600 inhabitants/km^2^	268	64	121	63.4	147	64.5
≥600 inhabitants/km^2^	151	36	70	36.6	81	35.5
**Lifestyle variables**						
Smoking						
Never or once	400	95.5	180	94.2	220	96.5
Less than daily	8	1.9	5	2.6	3	1.3
Daily	10	2.4	5	2.6	5	2.2
Missing	1	0.2	1	0.5	0	0
Alcohol use						
Never	263	62.8	128	67	135	59.2
<1 consumption monthly	93	22.2	37	19.4	56	24.6
Monthly or more	61	14.6	25	13.1	36	15.8
Missing	2	0.5	1	0.5	1	0.4
Active sports^a^						
Never or <1 x/week	63	15	26	13.6	37	16.2
1–2 times a week	152	36.3	59	30.9	93	40.8
>2 times a week	202	48.2	104	54.5	98	43
Missing	2	0.5	2	1	0	0
**Physical health characteristics**						
Body mass index^b^						
Underweight	34	8.1	16	8.4	18	7.9
Normal weight	302	72.1	147	77	155	68
Overweight, obese	83	19.8	28	14.7	55	24.1
Atopic disease^c^						
Yes	129	30.8	66	34.6	63	27.6
No	281	67.1	121	63.4	160	70.2
Missing	9	2.1	4	2.1	5	2.2
Infections (past year)						
Yes	244	58.2	110	57.6	134	58.8
No	162	38.7	76	39.8	86	37.7
Missing	13	3.1	5	2.6	8	3.5
**Mental health characteristics**						
Perceived stress (past 3 months)						
Low	113	27	73	38.2	40	17.5
Medium	164	39.1	76	39.8	88	38.6
High	139	33.2	39	20.4	100	43.9
Missing	3	0.7	3	1.6	0	0
Optimal vitality^d^						
Yes	56	13.4	27	14.1	29	12.7
No	360	85.9	161	84.3	199	87.3
Missing	3	0.7	3	1.6	0	0
Perceived happiness						
Low	34	8.1	7	3.7	27	11.8
High	382	91.2	181	94.8	201	88.2
Missing	3	0.7	3	1.6	0	0

*Data in numbers (n) and percentages (%), ^a^physical activity that causes sweating and/or heavy breathing, ^b^Body Mass Index classes based on age- and sex-specific Belgian growth curves, ^c^doctor-diagnosed asthma and/or atopic dermatitis and/or allergic rhinitis, ^d^vitality score > 77.4 (study population mean plus one standard deviation). EU, European Union; SDQ, Strengths and Difficulties Questionnaire*.

Hair cortisol concentrations are described in Table 2. The geometric mean HCC of all participants was 3.13 (95% CI: 2.90, 3.38) pg/mg, median HCC value was 2.98 pg/mg with an interquartile range of 2.28 pg/mg. Geometric mean HCC was higher for girls than boys [3.30 (95% CI: 2.99, 3.63) vs. 2.95 (95% CI: 2.61, 3.30) pg/mg], the difference was not statistically significant (*p* = 0.154).

**Table 2:** Descriptive statistics of hair cortisol concentrations of all participants and stratified by sex.

**Hair cortisol concentrations (pg/mg hair)**			
	**All** **(*****n*** **=** **419)**	**Boys** **(*****n*** **=** **191)**	**Girls** **(*****n*** **=** **228)**
10^th^ Percentile	1.53	1.41	1.65
25^th^ Percentile	2.10	1.88	2.32
Median	2.98	2.69	3.31
75^th^ Percentile	4.38	3.88	4.70
90^th^ Percentile	6.10	6.10	6.11
GM (95% CI)	3.13 (2.90, 3.38)	2.95 (2.61, 3.30)	3.30 (2.99, 3.63)

*CI, confidence interval; GM, geometric mean*.

### Assessment of Determinants of Hair Cortisol Concentrations in Adolescents

Table 3 presents results of associations between HCC and potential determinants of HCC in regression models, adjusted for sex and age. Results of the univariate analysis are presented in Supplementary Table 1. Effect modification of associations between potential determinants and HCC by sex is reported when significant.

**Table 3:** Linear regression analyses, adjusted for sex and age, for the assessment of determinants of hair cortisol concentrations in adolescents.

**Characteristic (n)**	**Linear regression adjusted for** **sex and age**	
	**Estimate (95% CI)**	***p*-value**
**Sociodemographic variables**		
Sex		
Boys (191)	reference	
Girls (228)	1.12 (0.96, 1.31)	0.138
Age (years)		0.221
[13.5–14.5] years (113)	reference	
[14.5–15.5] years (273)	1.07 (0.90, 1.28)	0.426
>15.5 years (33)	1.32 (0.97, 1.79)	0.083
Country of birth		0.356
Belgium (337)	reference	
EU (35)	0.86 (0.65, 1.14)	0.309
Outside EU (46)	1.12 (0.87, 1.44)	0.382
Household educational attainment		0.835
Primary (24)	reference	
Secondary (139)	1.11 (0.79, 1.55)	0.564
Tertiary (249)	1.08 (0.77, 1.50)	0.667
Equivalent income (monthly)		0.422
0–1250 Euro (89)	reference	
1251–1600 Euro (73)	1.09 (0.85, 1.39)	0.512
1601–2000 Euro (71)	0.99 (0.78, 1.27)	0.963
>2000 Euro (118)	0.89 (0.72, 1.11)	0.321
Perceived income adequacy		0.194
Difficult (119)	reference	
Rather easy (133)	0.91 (0.74, 1.11)	0.333
Easy to very easy (160)	0.84 (0.69, 1.01)	0.070
Area deprivation index		0.714
0–5.3% (103)	reference	
5.4–9.3% (105)	0.99 (0.79, 1.23)	0.397
9.4–15.5% (103)	1.04 (0.83, 1.29)	0.743
>15.5% (107)	1.11 (0.89, 1.38)	0.349
Urbanicity		0.177
Cities (57)	reference	
Towns and suburbs (303)	0.86 (0.68, 1.07)	0.177
Rural areas (59)	0.76 (0.57, 1.02)	0.064
Population density		
<600/km^2^ (268)	reference	
≥600/km^2^ (151)	**1.18 (1.00, 1.38)**	**0.047**
**Lifestyle variables**		
Smoking		0.967
Never or once (400)	reference	
Less than daily (8)	1.02 (0.58, 1.80)	0.932
Daily (10)	1.07 (0.64, 1.77)	0.804
Alcohol use		0.542
Never (263)	reference	
Less than monthly (93)	0.93 (0.77, 1.13)	0.466
Monthly or more (61)	1.08 (0.86, 1.36)	0.520
Active sports^a^		0.201
Never, occasionally (63)	reference	
1–2 times/week (152)	1.24 (0.98, 1.57)	0.073
>2 times/week (202)	1.17 (0.93, 1.47)	0.183
Hair washing frequency		0.766
Daily (67)	reference	
≥2 times a week (237)	0.96 (0.77, 1.20)	0.722
<2 times a week (114)	1.02 (0.80, 1.31)	0.847
Hair treatment		
No (409)	reference	
Yes (6)	0.72 (0.37, 1.38)	0.321
**Health characteristics**		
Body mass Index^b^		0.297
Normal weight (302)	reference	
Underweight (34)	0.89 (0.67, 1.19)	0.433
Overweight, obese (83)	1.13 (0.93, 1.37)	0.226
Waist circumference		0.298
≤ 68 cm (101)	reference	
68–72 cm (109)	1.11 (0.89, 1.38)	0.342
72–78 cm (101)	1.19 (0.95, 1.49)	0.121
>78 cm (106)	1.21 (0.98, 1.51)	0.082
Infections (past year)		
No (162)	reference	
Yes (244)	0.94 (0.80, 1.10)	0.450
Atopic disease^c^
No (281)	reference	
Yes (129)	1.17 (0.99, 1.38)	0.071
Corticosteroid medication		
No (409)	reference	
Yes (8)	0.92 (0.52, 1.62)	0.771
Menarcheal status (girls)
Pre-menarche (23)	reference	
Post-menarche (205)	**1.61 (1.17, 2.23)**	**0.004**
Oral contraceptives (girls)		
No (189)	reference	
Yes (39)	1.01 (0.78, 1.32)	0.927
SDQ Total difficulties		0.714
Normal (0–15) (325)	reference	
Borderline (16–19) (57)	0.99 (0.79, 1.24)	0.915
Abnormal (20–40) (35)	1.12 (0.84, 1.50)	0.430
SDQ Prosocial scale		0.592
Normal (6–10) (381)	reference	
Borderline (5) (22)	1.12 (0.80, 1.59)	0.509
Abnormal (0–4) (14)	0.85 (0.55, 1.31)	0.457
Perceived stress		0.382
Low (113)	reference	
Medium (164)	1.05 (0.87, 1.28)	0.607
High (139)	0.92 (0.75, 1.13)	0.449
Optimal vitality^d^		
No (360)	reference	
Yes (56)	**0.76 (0.61, 0.95)**	**0.015**
Perceived happiness		
Low (34)	reference	
High (382)	0.99 (0.74, 1.31)	0.918
Preterm birth (<37 weeks)		
No (387)	reference	
Yes (25)	1.07 (0.77, 1.48)	0.704
Low birth weight (<2.5 kg)		
No (372)	reference	
Yes (24)	0.94 (0.67, 1.33)	0.735
Maternal smoking during pregnancy		
No (354)	reference	
Yes (58)	1.02 (0.82, 1.28)	0.833
**Meteorology**		
3-month average temperature		0.459
<6°C (148)	reference	
6–12°C (179)	0.98 (0.82, 1.17)	0.836
>12°C (92)	1.12 (0.91, 1.38)	0.301
3-month average UV radiation		0.659
<300 J/m^2^ (137)	reference	
300–1000 J/m^2^ (123)	0.98 (0.80, 1.20)	0.855
>1000 J/m^2^ (159)	1.07 (0.89, 1.28)	0.498
Season of sampling		0.633
Winter (136)	reference	
Spring (186)	1.01 (0.84, 1.21)	0.921
Summer (0)	-	-
Fall (97)	1.10 (0.89, 1.36)	0.370
**Effect modification by sex**		
Urbanicity x Sex		**0.011**
Population density x Sex		**0.024**

*Estimates (β) are presented with their 95% confidence intervals (95% CI) as the factor change in HCC compared to the HCC of the reference category or to the opposite situation for dichotomous (yes/no) variables. Significant associations are marked in bold. ^a^physical activity that causes sweating and/or heavy breathing, ^b^Body Mass Index classes based on age- and sex-specific Belgian growth curves, ^c^doctor-diagnosed asthma and/or atopic dermatitis and/or allergic rhinitis, ^d^vitalityscore above the cut-off value of study population mean plus one standard deviation. EU, European Union; SES, socioeconomic status; SDQ, Strengths and Difficulties Questionnaire*.

#### Sociodemographic Determinants of HCC

We did not observe a significant association between HCC and sex *(p* = 0.138) nor between HCC and age (*p* = 0.221). HCC was not significantly associated with adolescents' country of birth (*p* = 0.356). There were no significant associations between HCC and household educational attainment (*p* = 0.835) or equivalent income *(p* = 0.422). Overall, HCC was not significantly associated with perceived income adequacy (*p* = 0.194). Between perceived income adequacy categories, we observed a tendency toward significantly lower HCC in adolescents, living in households that reported having it easy make ends meet, compared to adolescents, living in households that reported having it difficult to make ends meet (*p* = 0.070). The model estimated HCC of adolescents from households that reported having it easy to make ends meet on average a factor 0.84 (95% CI: 0.69, 1.01) lower, compared to their counterparts from households that reported having it difficult to make ends meet.

Living in a more densely populated neighborhood was positively associated with adolescents' HCC [*p* = 0.047, β = 1.18 (95% CI: 1.00, 1.38)]. The association between neighborhood population density and HCC was significantly modified by sex and was driven by boys (*p*-interaction = 0.024). Boys who lived in a densely populated neighborhoods, had significantly higher HCC compared to their peers from less densely populated neighborhoods [*p* = 0.002, β = 1.44 (95% CI: 1.14, 1.81)]. The association was not significant in girls [*p* = 0.934, β = 0.99 (95% CI: 0.80, 1.23)]. HCC for all adolescents taken together, was not significantly associated with neighborhood urbanicity (*p* = 0.177). However, this association was also significantly modified by sex (*p*-interaction = 0.011). Boys, living in towns/suburbs or in rural areas had significantly lower HCC compared to boys that lived in cities [*p* = 0.002, β = 0.56 (95% CI: 0.38, 0.80) and *p* = 0.002, β = 0.44 (95% CI: 0.27, 0.74), respectively]. HCC was not significantly associated with urbanicity in girls [*p* = 0.521, β = 1.10 (95% CI: 0.83, 1.46) and *p* = 0.980, β = 1.00 (95% CI: 0.70, 1.41) for towns/suburbs and for rural areas, respectively, compared to cities].

#### Lifestyle, Health and Meteorological Determinants of HCC

We did not observe significant associations of HCC with lifestyle characteristics. We found no significant associations of HCC with anthropometric measures or perinatal factors. The association between HCC and atopic disease tended toward significance [*p* = 0.071, β = 1.17 (95% CI: 0.99, 1.38)]. In girls, HCC was significantly higher in post-menarcheal girls compared to pre-menarcheal girls in an age-adjusted model [*p* = 0.004, β = 1.61 (95% CI: 1.17, 2.23)]. HCC was not significantly associated with mental health variables such as SDQ scores, perceived stress or general happiness. Adolescents reporting optimal vitality, however, had significantly lower HCC than adolescents that did not score optimal on vitality [*p* = 0.015, β = 0.76 (95% CI: 0.61, 0.95)]. There were no significant associations of HCC with meteorological factors or season of sampling.

#### Determinants of HCC: Accounting for Multiple Simultaneous Determinants

In the next step, significant determinants of HCC were evaluated in a multiple regression model, adjusted with all variables that showed a significance level ≤ 0.2 in the determinant assessment. Spearman rank correlations between all variables that met this criterion were first evaluated, results are presented in Supplementary Table 2. We observed a weak negative correlation between optimal vitality and atopic disease (Spearman rank ρ = −0.131), a weak positive correlation between age and menarcheal status (Spearman rank ρ = 0.166) and a moderately strong positive correlation between neighborhood urbanicity and population density (Spearman rank ρ = 0.545). Adolescents' HCC remained negatively associated with optimal vitality [*p* = 0.035, β = 0.78 (95% CI: 0.63, 0.98)] and population density [*p* = 0.036, β = 1.19 (95% CI: 1.04, 1.40)] after adjustment for sex, age, perceived income adequacy and atopic disease. The model, presented in Supplementary Table 3, explained 3% of the variance in adolescents' HCC. In this model, the association between HCC and atopic disease further attenuated [*p* = 0.232, β = 1.11 (95% CI: 0.94, 1.31)]. HCC was overall not significantly associated with perceived income adequacy (*p* = 0.120): we however observed significant lower HCC in adolescents living in a household that reported having it easy to get by with their income, compared to adolescents, living in households that reported having it difficult to get by [*p* = 0.040, β = 0.82 (95% CI: 0.67, 0.99)].

**Figure 1:**
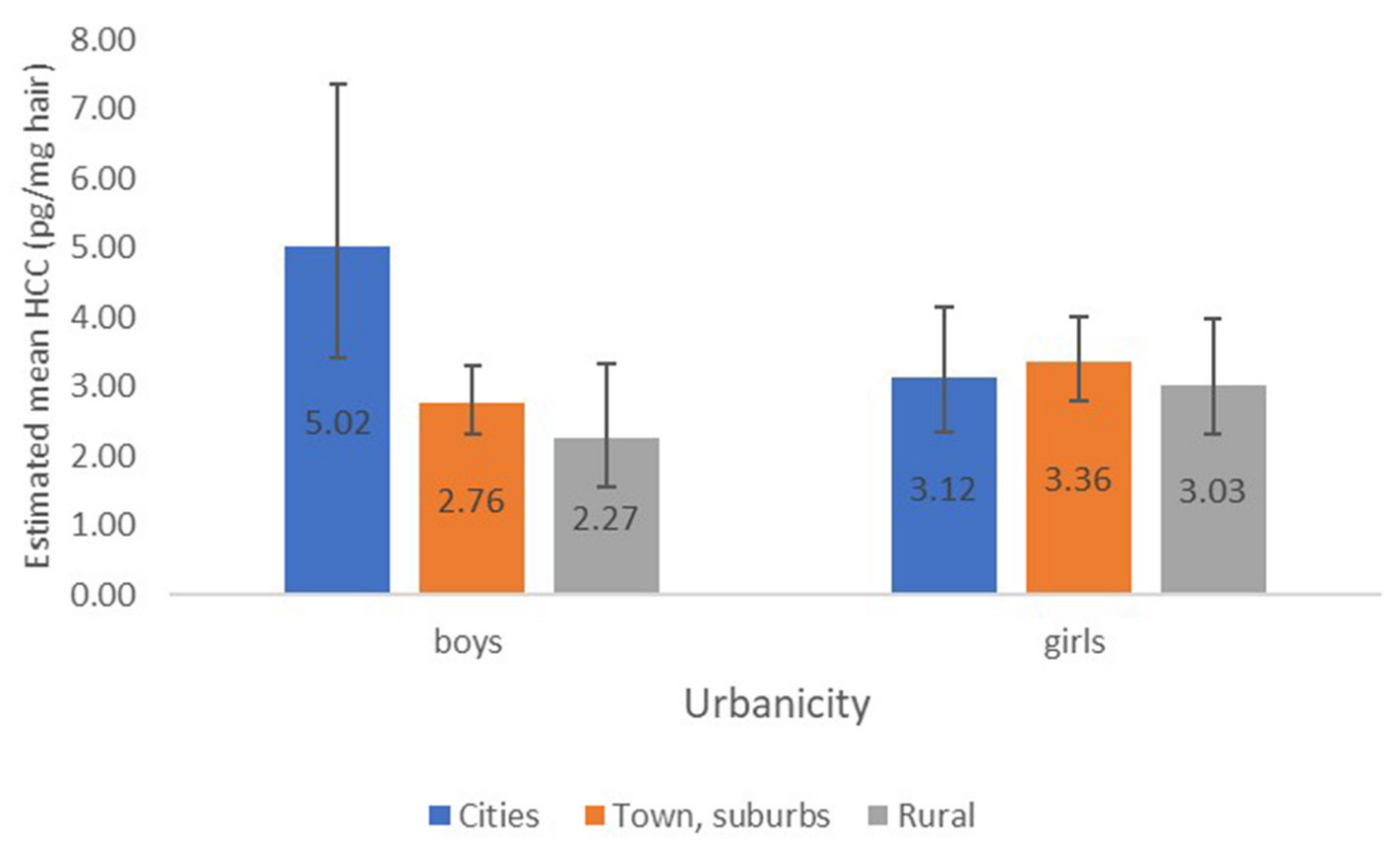
Estimated mean HCC (±95% CI) in relation to urbanicity for boys and girls, associations of HCC with urbanicity adjusted for age, perceived income adequacy, atopic disease and optimal vitality.

As population density is a criterion for urbanicity, both variables were assessed in separate models. When the interaction term of sex and population density was additionally added to the model, as presented in Supplementary Table 4, HCC of boys from a densely populated neighborhood remained significantly higher compared to HCC of those from less densely populated neighborhoods [*p* = 0.002, β = 1.46 (95% CI: 1.15, 1.85)] after adjustment for age, perceived income adequacy, atopic disease and optimal vitality. The model explained 4.1% in boys' variation in HCC. When the interaction term of sex and neighborhood urbanicity was included in the model, the model explained an equal proportion of 4.1% of variations in HCC of adolescent boys. Boys from towns/suburbs or rural areas exhibited significantly lower HCC compared to boys from cities [*p* = 0.002, β = 0.55 (95% CI: 0.38, 0.81) and *p* = 0.003, β = 0.45 (95% CI: 0.27, 0.76), respectively]. The estimated mean HCC for boys and girls in relation to urbanicity are illustrated in Figure 1.

Menarcheal status remained a significant determinant of HCC in girls [*p* = 0.006, β = 1.59 (95% CI: 1.14, 2.23)] after adjustment for age, perceived income adequacy, population density, optimal vitality and atopic disease. The model, presented in Supplementary Table 5, explained 3.5% of variations in adolescent girls' HCC.

#### Sensitivity Analysis

In a sensitivity analysis, linear regressions analyses for the assessment of determinants of HCC were performed using winsorized data. In accordance with literature (8, 9, 40, 51), we observed several outliers in our data. Three participants exhibited HCC > 3 standard deviations (SD) from the mean and were winsorized to the mean + 3 SD. Results of the analyses are presented in Supplementary Table 6, winsorizing the data was of little influence on the observed associations and interactions. We found similar significant differences by sex for the association of neighborhood population density with HCC (*p*-interaction = 0.022), with significantly higher HCC for boys from densely populated neighborhoods [*p* = 0.003, β = 1.39 (95% CI: 1.12, 1.73)], but no significant difference in HCC by population density was found for girls [*p* = 0.989, β = 1.00 (95% CI: 0.82, 1.23)]. We also observed similar differences by sex for the association of urbanicity with HCC (*p*-interaction = 0.014). Boys that lived in towns and suburbs or rural areas had significantly lower HCC compared to boys that lived in cities [*p* = 0.003, β = 0.59 (95% CI: 0.42, 0.83) and *p* = 0.002, β = 0.47 (0.29, 0.76), respectively]. There was no significant difference in HCC across urbanicity for girls.

We reinterpreted our results after controlling for the false discovery rate (FDR) at *q* = 0.10 using the Benjamini and Hochberg method. Optimal vitality did not remain a significant determinant of HCC when we controlled for the FDR at *q* = 0.10, while population density, urbanicity remained significant determinants of adolescent boys' HCC and menarcheal status remained a significant determinant of adolescent girls' HCC.

## Discussion

In this study, we explored potential determinants of chronic biological stress in a general population of Flemish adolescents, using hair cortisol concentrations as a novel objective biomarker of chronic stress. HCC was significantly lower in adolescents with an optimal vitality, an indicator of a positive mental health status, and significantly higher in adolescents from densely populated neighborhoods. The association of HCC with population density was driven by boys. Moreover, boys from urban neighborhoods had significantly higher HCC than those from towns, suburbs and rural areas. The associations remained significant after adjustment for age, perceived income adequacy, atopic disease and optimal vitality. This is the first study to identify neighborhood urbanicity as a determinant of chronic stress in a general population of adolescents. We identified post-menarcheal status as an important determinant of HCC in girls.

We did not observe a significant difference in HCC between adolescent boys and girls in this study. Our results are in line with a large-scale multi-country study that measured hair cortisol in adolescents using LC/MS-MS (12–17 years, *n* = 353) (51). Other studies, including children and adolescents, found a significant difference in HCC by sex (7). This difference seems to be predominantly present in childhood. A recent study (*n* = 434) observed significantly higher HCC in boys compared to girls in prepuberty, HCC values in both sexes converged after the onset of puberty (13). Similar to other recent adolescent-specific studies (10, 51), age was not a significant HCC determinant in this study. Research in broader age ranges did find significant associations of HCC with age (7, 13).

In line with previous research in adolescent populations (52, 53), we did not observe a significant relation between household educational attainment and HCC. The household equivalent income was not significantly associated with HCC. Adolescents from households that reported having it easy to very easy to get by with their income exhibited borderline significantly lower HCC compared to adolescents from households that reported having it difficult to make ends meet. Previous research has indicated that subjective income adequacy measures exhibit a positive association with health and well-being, above and beyond the health benefits associated with objective income measures (3).

In contrast to Wagner et al. (13) in the age group of 5–18 years (*n* = 434) (13), we found no significant associations between perinatal factors (low birth weight, preterm birth) and adolescents' HCC. Possibly, the associations attenuate over time, a prospective cohort study found maternal prenatal smoking and low birth weight to be associated with HCC in children, however, the associations did not persist in adolescence (12). We identified post-menarcheal status as an important determinant of HCC in girls. The onset of menstruation is a hallmark of female pubertal development that is associated with a marked increase in cortisol secretion (54). In accordance with previous research (55), we found no significant association of girls' contraceptive use with HCC. We observed a tendency toward a significant association of HCC with atopic diseases, which are known to be widespread among adolescents. HCC was not significantly associated with psychosocial variables such as SDQ scores or perceived stress. These results are consistent with existing literature (7, 10, 56). Adolescents may experience psychological stress when they are not stressed biologically and alternatively may be biologically stressed, but not report any psychological stress (18). Optimal vitality was significantly associated with lower HCC in our study. An exploratory study previously assessed HCC in relation to a positive mental health status in adolescents (12–18 years, *n* = 27) and observed an inverse association between HCC and optimism (18). According to the World Health Organization, mental health is a state of well-being in which an individual realizes his or her own abilities, can cope with the normal stresses of life and is able to make a contribution to his or her community (57). Optimal vitality did not remain a significant determinant when we controlled for multiple testing. However, our aim was to identify potential determinants of adolescents' HCC. We therefore still consider optimal vitality as a potential determinant of HCC. Possibly, vitality and coping strategies play a role in associations between stressors and biological stress. We therefore suggest future research to consider optimal vitality as a determinant of HCC in adolescence.

The observed association of HCC with a high neighborhood population density in all adolescents was driven by boys. Furthermore, urbanicity was a significant determinant of HCC in boys in this study, but not in girls. Sexually dimorphic HPA axis reactivity has previously been reported, with men showing a greater cortisol response to real-life or controlled laboratory stress compared to women (58). Flanders, our study region, is known as one of the most densely populated areas in Europe (59). Moreover, urbanization is one of the dominant demographic trends in the 21^st^ century (22). Importantly, living in an urban area seems to be associated with an increased risk of mental health problems, compared to living in a rural area (19). Retrospective studies showed that this increased risk for mental health problems in adults may be greater for those who grew up in an urban context (60, 61). Residential urbanicity has also been associated with adverse cardio-metabolic health outcomes (62, 63). Despite the evidence of a higher incidence of health problems in urban areas, the mechanisms underlying this association are not well understood (22). Extensive research has put forward several pathways through which an urban environment may affect physical and mental health, including the socioeconomic circumstances and social context of an urban residential environment and environmental challenges such as air pollution, noise, heat and lack of greenspace (21, 22). A Flemish study, tracking 175 children during a 3-year time period between childhood and adolescence, found that higher residential exposure to semi-natural and forested areas was associated with increased feelings of happiness, while a poorer emotional status was seen with increased residential traffic exposure (56). Chronic stress through HPA axis activation has been suggested as a potential biological mechanism, underlying the association between urbanicity and health (19). In our adolescent study population, household SES and neighborhood SES were not associated with HCC, nor was psychosocial stress. Possibly, environmental factors such as air pollution and noise play a role. Animal and human studies, using salivary and serum cortisol as short-term markers of HPA axis activity, have demonstrated that air pollutants may dose-dependently increase the release of cortisol (64). Increased salivary cortisol levels have also been associated with noise exposure (65). In a recent study in Flanders, we observed a positive association between residential proximity to major roads and HCC in pregnant women (66). Further research in this study population may elucidate the relationship between air pollution, noise and participants' HCC.

This study has several strengths. First, all hair samples were collected by a small team of trained nurses, ensuring low variability in sampling method across our study population. We used a sensitive LC-MS/MS method to measure hair cortisol, as recommended. The multidisciplinary setup of the FLEHS-4 study, resulted in detailed information on physical and mental health, social and behavioral factors, as well as geographical information. This enabled us to characterize a broad range of variables that have previously been postulated as potential determinants of HCC in literature, but have not yet been explored in adolescents. We also need to address several limitations. The bidirectional relationship between stress and health outcomes makes cross-sectional studies vulnerable to reverse causation (12). Longitudinal follow-up of our study population could provide more insight in direction of associations between HCC and health. We had no information on natural hair color, which has been hypothesized to influence HCC, but previous studies found no associations of HCC with natural hair color in adolescents (7, 11). We collected information on corticosteroid medication use in a period of 14 days prior to sampling. Information on less recent medication use was not collected. We did not observe significant associations of HCC with local ambient temperature; however, participants were not examined during the warmer summer season because of school holidays. Our aim was to identify potential determinants of adolescents' HCC that need to be confirmed or underpinned in future research. We therefore assessed a broad range of potential determinants of HCC. Although we evaluated our results after correcting for multiple testing, we cannot rule out the possibility of observing significant associations due to chance. More targeted assessment of health, psychosocial variables, behavior and urban environmental exposures could help future, larger, studies to gain a deeper insight in the determinants of adolescents' chronic stress. Growing attention is currently being paid to concept of the urban exposome, the totality of environmental exposures and their endogenous response in shaping disease risk and disease development of urban dwellers (67, 68). Applying the exposome concept to stress research can provide a more holistic view of all factors that contribute to chronic stress. The emerging technology of wearable sensors can help to easily and rapidly generate the necessary information (22).

In conclusion, our results suggest that personal factors as well as residential environment significantly determine hair cortisol concentrations in adolescents, a marker of chronic biological stress. Preventing chronic stress, a known risk factor for ill health, should therefore not be limited to the personal level but should also include preventive measures at the neighborhood level. This new knowledge may support future stress research and evidence-informed public health.

## Data Availability Statement

The datasets presented in this article are not readily available because access to the data can only be granted by the data owner. Requests to access these datasets should be directed to VITO, FLEHS.datamanagement@vito.be.

## Ethics Statement

The studies involving human participants were reviewed and approved by Antwerp University Hospital. Written informed consent to participate in this study was provided by the participants' legal guardian/next of kin.

## Author Contributions

VV, SR, LB, IL, ED, VN, SD, NV, AdC, CT, TN, and GS have made substantial contributions to the conception and design of the study. VV, AnC, EG, GK, LM, LB, AD, CF, DC, EB, SV, and ED contributed to acquisition of data and statistical analysis. FN contributed to design of the study, acquisition of data and biomarker analysis. VV, SR, LB, BM, and GS participated in the interpretation of the data. VV drafted the manuscript. SR and GS helped to draft the manuscript. VV, SR, EG, AnC, GK, LM, FN, LB, EB, SV, BM, DC, IL, AD, CF, ED, VN, SD, AdC, NV, CT, TN, and GS were involved in critically reviewing and editing the manuscript. GS coordinated the Flemish Environment and Health Study. All authors approved the manuscript to be published.

## Funding

This paper is based on research conducted within the framework of the Flemish Center of Expertise on Environment and Health (FLEHS 2016-2020), funded by the Government of Flanders, Department of Environment & Spatial Development. VV was supported by a PhD fellowship at the University of Antwerp and VITO, funded by the Flemish Center of Expertise on Environment and Health.

## Author Disclaimer

The views expressed herein are those of the author(s) and are not necessarily endorsed by the government of Flanders.

## Conflict of Interest

The authors declare that the research was conducted in the absence of any commercial or financial relationships that could be construed as a potential conflict of interest.

## Publisher's Note

All claims expressed in this article are solely those of the authors and do not necessarily represent those of their affiliated organizations, or those of the publisher, the editors and the reviewers. Any product that may be evaluated in this article, or claim that may be made by its manufacturer, is not guaranteed or endorsed by the publisher.
