# Supplementary material for: Determinants of Chronic Biological Stress, Measured as Hair Cortisol Concentration, in a General Population of Adolescents: From Individual and Household Characteristics to Neighborhood Urbanicity
Source: Front Public Health. 2021 Nov 23;9:669022. doi: 10.3389/fpubh.2021.669022 (PMC8650704; doi:10.3389/fpubh.2021.669022)
Supplement: Supplementary file 1 [file Data_Sheet_1.docx]

Supplementary Material

Determinants of chronic biological stress, measured as hair cortisol concentration, in a general population of adolescents: from individual and household characteristics to neighborhood urbanicity

**Veerle J Verheyen^1,2*^, Sylvie Remy^1^, Eva Govarts^1^, Ann Colles^1^, Gudrun Koppen^1^, Laura Rodriguez Martin^1^, Flemming Nielsen^3^, Liesbeth Bruckers^4^, Esmée M Bijnens^5^, Stijn Vos^5^, Bert Morrens^6^, Dries Coertjens^6^, Ilse Loots^6^, Annelies De Decker^7^, Carmen Franken^7^, Elly Den Hond^7^, Vera Nelen^7^, Stefaan De Henauw^8^, Adrian Covaci^9^, Nicolas van Larebeke^10,11^, Caroline Teughels^12^, Tim S Nawrot^5^, Greet Schoeters^1,2^**

**Table S1. Univariate linear regression analyses for the assessment of determinants of hair cortisol concentrations in adolescents**

| **Characteristic (n)** | **Unadjusted linear regression** | |
| --- | --- | --- |
|  |  |  |
|  | **Estimate (95% CI)** | ***p*-Value** |
| **Sociodemographic variables** | | |
| Sex |  |  |
| Boys (191) | reference |  |
| Girls (228) | 1.12 (0.96, 1.30) | 0.154 |
| Age |  | 0.240 |
| [13.5 - 14.5] years (113) | reference |  |
| [14.5 - 15.5] years (273) | 1.07 (0.90, 1.27) | 0.458 |
| > 15.5 years (33) | 1.31 (0.96, 1.78) | 0.092 |
| Country of birth |  | 0.295 |
| Belgium (337) | reference |  |
| EU (35) | 0.90 (0.68, 1.18) | 0.444 |
| Outside EU (46) | 1.17 (0.92, 1.50) | 0.203 |
| Household educational attainment |  | 0.774 |
| Primary (24) | reference |  |
| Secondary (139) | 1.06 (0.76, 1.49) | 0.726 |
| Tertiary (249) | 1.00 (0.72, 1.39) | 0.989 |
| Equivalent income (monthly) |  | 0.328 |
| 0 - 1250 Euro (89) | reference |  |
| 1251 - 1600 Euro (73) | 1.08 (0.85, 1.38) | 0.527 |
| 1601 - 2000 Euro (71) | 0.99 (0.77, 1.27) | 0.923 |
| > 2000 Euro (118) | 0.88 (0.71, 1.09) | 0.24 |
| Perceived income adequacy |  | 0.144 |
| Difficult (119) | reference |  |
| Rather easy (133) | 0.90 (0.74, 1.09) | 0.283 |
| Easy to very easy (160) | **0.83 (0.68, 1.00)** | **0.049** |
| Area deprivation index |  | 0.743 |
| 0 - 5.3 % (103) | reference |  |
| 5.4 - 9.3 % (105) | 0.98 (0.79, 1.22) | 0.865 |
| 9.4 - 15.5 % (103) | 1.03 (0.83, 1.29) | 0.768 |
| >15.5 % (107) | 1.10 (0.88, 1.37) | 0.390 |
| Urbanicity |  | 0.184 |
| Cities (57) | reference |  |
| Towns and suburbs (303) | 0.84 (0.67, 1.05) | 0.129 |
| Rural areas (59) | 0.77 (0.57, 1.03) | 0.075 |
| Population density |  |  |
| <600 /km^2^ (268) | reference |  |
| ≥600/km^2^ (151) | **1.18 (1.00, 1.38)** | **0.045** |
| **Lifestyle variables** | | |
| Smoking |  | 0.898 |
| Never or once (400) | reference |  |
| Less than daily (8) | 1.03 (0.59, 1.81) | 0.911 |
| Daily (10) | 1.12 (0.68, 1.86) | 0.651 |
| Alcohol use |  | 0.413 |
| Never (263) | reference |  |
| Less than monthly (93) | 0.95 (0.79, 1.15) | 0.598 |
| Monthly or more (61) | 1.13 (0.90, 1.41) | 0.283 |
| Active sports^a^ |  | 0.227 |
| Never, occasionally (63) | reference |  |
| 1 - 2 times /week (152) | 1.23 (0.97, 1.55) | 0.088 |
| > 2 times /week (202) | 1.13 (0.90, 1.42) | 0.279 |
| Hair washing frequency |  | 0.822 |
| Daily (67) | reference |  |
| ≥ 2 times a week (237) | 0.99 (0.80, 1.23) | 0.929 |
| < 2 times a week (114) | 1.05 (0.82, 1.33) | 0.705 |
| Hair treatment (6) |  |  |
| No (409) | reference |  |
| Yes (6) | 0.81 (0.42, 1.54) | 0.512 |
| **Health characteristics** | | |
| Body mass Index^b^ |  | 0.227 |
| Normal weight (302) | reference |  |
| Underweight (34) | 0.90 (0.68, 1.20) | 0.474 |
| Overweight, obese (83) | 1.15 (0.95, 1.40) | 0.148 |
| Waist circumference |  | 0.305 |
| ≤ 68 cm (101) | reference |  |
| [68 - 72 cm] (109) | 1.11 (0.89, 1.38) | 0.346 |
| [72 - 78 cm] (101) | 1.18 (0.95, 1.47) | 0.142 |
| >78 cm (106) | 1.22 (0.98, 1.52) | 0.077 |
| Infections (past year) |  |  |
| No (162) | reference |  |
| Yes (244) | 0.95 (0.81, 1.12) | 0.549 |
| Atopic disease^c^ |  |  |
| No (281) | reference |  |
| Yes (129) | 1.17 (0.99, 1.38) | 0.072 |
| Corticosteroid medication |  |  |
| No (409) | reference |  |
| Yes (8) | 0.90 (0.51, 1.57) | 0.705 |
| Menarcheal status (girls) |  |  |
| Pre-menarche (23) |  |  |
| Post-menarche (205) | **1.65 (1.20, 2.26)** | **0.002** |
| Oral contraceptives (girls) |  |  |
| No (189) |  |  |
| Yes (39) | 1.05 (0.81, 1.36) | 0.722 |
| SDQ Total difficulties |  | 0.435 |
| Normal (0-15) (325) | reference |  |
| Borderline (16-19) (57) | 1.02 (0.81, 1.27) | 0.883 |
| Abnormal (20-40) (35) | 1.20 (0.91, 1.59) | 0.197 |
| SDQ Prosocial scale |  | 0.695 |
| Normal (6-10) (381) | reference |  |
| Borderline (5) (22) | 1.11 (0.79, 1.57) | 0.541 |
| Abnormal (0-4) (14) | 0.88 (0.58, 1.36) | 0.571 |
| Perceived stress |  | 0.633 |
| Low (113) | reference |  |
| Medium (164) | 1.07 (0.88, 1.30) | 0.483 |
| High (139) | 0.99 (0.81, 1.20) | 0.895 |
| Optimal vitality^d^ |  |  |
| No (360) | reference |  |
| Yes (56) | **0.75 (0.60, 0.93)** | **0.010** |
| Perceived happiness |  |  |
| Low (34) | reference |  |
| High (382) | 0.92 (0.70, 1.22) | 0.560 |
| Preterm birth (< 37 weeks) |  |  |
| No (387) | reference |  |
| Yes (25) | 1.04 (0.75, 1.43) | 0.834 |
| Low birth weight (<2.5 kg) |  |  |
| No (372) | reference |  |
| Yes (24) | 0.98 (0.69, 1.39) | 0.914 |
| Maternal smoking during pregnancy |  |  |
| No (354) | reference |  |
| Yes (58) | 1.05 (0.84, 1.31) | 0.677 |
| **Meteorology** | | |
| 3-month average temperature |  | 0.462 |
| <6 °C (148) | reference |  |
| 6 – 12 °C (179) | 0.99 (0.83, 1.18) | 0.883 |
| >12 °C (92) | 1.12 (0.91, 1.37) | 0.303 |
| 3-month average UV radiation |  | 0.75 |
| <300 J/m^2^ (137) | reference |  |
| 300 – 1000 J/m^2^ (123) | 0.99 (0.82, 1.21) | 0.946 |
| > 1000 J/m^2^ (159) | 1.06 (0.88, 1.27) | 0.536 |
| Season of sampling |  | 0.587 |
| Winter (136) | reference |  |
| Spring (186) | 1.01 (0.85, 1.21) | 0.895 |
| Summer (0) | - | - |
| Fall (97) | 1.11 (0.90, 1.36) | 0.339 |

Estimates (β) are presented with their 95% confidence intervals (95%CI) as the factor change in HCC compared to the HCC of the reference category or to the opposite situation for dichotomous (yes/no) variables. Significant associations are marked in bold. ^a^physical activity that causes sweating and/or heavy breathing, ^b^Body Mass Index classes based on age- and sex-specific Belgian growth curves, ^c^doctor-diagnosed asthma and/or atopic dermatitis and/or allergic rhinitis^, d^cut-off value of study population mean plus one standard deviation. Abbreviations: EU European Union, SES socioeconomic status, SDQ Strengths and Difficulties Questionnaire

**Table S2. Spearman rank correlations between variables, included in multiple linear regression models of HCC in relation to potential determinants in sensitivity analysis**

|  | **Age** | **Sex** | **Perceived income adequacy** | **Atopic disease** |  | **Optimal vitality** | **Urbanicity** | **Population density** | **Menarcheal status** |
| --- | --- | --- | --- | --- | --- | --- | --- | --- | --- |
| **Age** | 1.000 | -0.039 | **-0.122** | 0.025 |  | -0.066 | -0.021 | 0.066 | **0.166** |
| **Sex** |  | 1.000 | 0.029 | -0.047 |  | -0.024 | -0.036 | -0.012 | - |
| **Perceived income adequacy** |  |  | 1.000 | 0.030 |  | 0.054 | 0.070 | 0.031 | -0.022 |
| **Atopic disease** |  |  |  | 1.000 |  | **-0.131** | 0.049 | 0.077 | -0.017 |
| **Optimal vitality** |  |  |  |  |  | 1.000 | -0.088 | -0.016 | -0.047 |
| **Urbanicity** |  |  |  |  |  |  | 1.000 | **0.545** | -0.012 |
| **Population density** |  |  |  |  |  |  |  | 1.000 | 0.066 |
| **Menarcheal status** |  |  |  |  |  |  |  |  | 1.000 |

Significant correlations ( p-value ≤ 0.05 ) are presented bold

**Table S3. Multiple linear regression analysis for the assessment of hair cortisol concentrations in Flemish adolescents, both sexes included**

| **HCC** | **(n )Variable** | **β (95%CI)** | ***p*-value** |
| --- | --- | --- | --- |
|  | **Sex** |  | 0.133 |
|  | (182) Boy | reference |  |
|  | (220) Girl | 1.13 (0.96, 1.32) | 0.133 |
|  | **Age** |  | 0.491 |
|  | (110) [13.5 - 14.5] | reference |  |
|  | (261) [14.5 - 15.5] | 1.04 (0.87, 1.24) | 0.684 |
|  | (31) > 15.5 | 1.21 (0.88, 1.67) | 0.234 |
|  | **Perceived income adequacy** |  | 0.120 |
|  | (116) Difficult | reference |  |
|  | (131) Rather easy | 0.90 (0.74, 1.10) | 0.295 |
|  | (155) Easy to very easy | 0.82 (0.67, 0.99) | 0.040 |
|  | **Population density** |  | **0.036** |
|  | (257) < 600 inhabitants/km^2^ | reference |  |
|  | (145) ≥600 inhabitants/km^2^ | 1.19 (1.04, 1.40) | 0.036 |
|  | **Atopic disease** |  | 0.232 |
|  | (277) No | reference |  |
|  | (125) Yes | 1.11 (0.94, 1.31) | 0.232 |
|  | **Optimal vitality** |  | **0.035** |
|  | (346) No | reference |  |
|  | (56) Yes | 0.78 (0.63, 0.98) | 0.035 |
| R^2^ = 0.030 | |  |  |

**Table S4. Multiple linear regression analysis for the assessment of hair cortisol concentrations in Flemish adolescents, including effect modification by sex**

| **Model including population density** | | | |
| --- | --- | --- | --- |
| **HCC** | **(n) Variable** | **β (95%CI)** | ***p*-value** |
|  | **Sex** |  | 0.414 |
|  | (182) Boy | reference |  |
|  | (220) Girl | 1.07 (0.91, 1.26) | 0.414 |
|  | **Age** |  | 0.467 |
|  | (110) [13.5, 14.5] | reference |  |
|  | (261) [14.5, 15.5] | 1.05 (0.88, 1.25) | 0.609 |
|  | (31) > 15.5 | 1.22 (0.89, 1.68) | 0.217 |
|  | **Perceived income adequacy** |  | 0.148 |
|  | (116) Difficult | reference |  |
|  | (131) Rather easy | 0.89 (0.73, 1.08) | 0.248 |
|  | (155) Easy to very easy | 0.83 (0.68, 1.00) | 0.051 |
|  | **Population density** |  | **0.022** |
|  | (257) < 600 inhabitants/km^2^ | reference |  |
|  | (145) ≥600 inhabitants/km^2^ | 1.21 (1.03, 1.42) | 0.022 |
|  | **Atopic disease** |  | 0.207 |
|  | (277) No | reference |  |
|  | (125) Yes | 1.11(0.94, 1.32) | 0.207 |
|  | **Optimal vitality** |  | **0.037** |
|  | (346) No | reference |  |
|  | (56) Yes | 0.79 (0.63, 0.99) | 0.037 |
|  | **Sex x population density** |  | **0.024** |
|  | Population density boys | 1.46 (1.15, 1.85) | **0.002** |
|  | Population density girls | 1.00 (0.80, 1.25) | 0.984 |
| R^2^ = 0.041 |  |  |  |

| **Model including urbanicity** | | | |
| --- | --- | --- | --- |
| **HCC** | **(n )Variable** | **β (95%CI)** | **p-value** |
|  | **Sex** |  | 0.977 |
|  | (182) Boy | reference |  |
|  | (220) Girl | 1.00 (0.81, 1.25) | 0.977 |
|  | **Age** |  | 0.436 |
|  | (110) [13.5, 14.5] | reference |  |
|  | (261) [14.5, 15.5] | 1.07 (0.90, 1.28) | 0.448 |
|  | (31) > 15.5 | 1.22 (0.89, 1.68) | 0.209 |
|  | **Perceived income adequacy** |  | 0.075 |
|  | (116) Difficult | reference |  |
|  | (131) Rather easy | 0.87 (0.72, 1.07) | 0.183 |
|  | (155) Easy to very easy | 0.80 (0.66, 0.97) | 0.023 |
|  | **Urbanicity** |  | **0.032** |
|  | Cities | reference |  |
|  | Towns, suburbs | 0.77 (0.61, 0.98) | 0.031 |
|  | Rural | 0.66 (0.48, 0.91) | 0.011 |
|  | **Atopic disease** |  | 0.203 |
|  | (277) No | reference |  |
|  | (125) Yes | 1.12 (0.94, 1.32) | 0.203 |
|  | **Optimal vitality** |  | 0.099 |
|  | (346) No | reference |  |
|  | (56) Yes | 0.83 (0.66, 1.04) | 0.099 |
|  | **Sex x urbanicity** |  | **0.017** |
|  | Boys cities | reference |  |
|  | Boys towns/suburbs | 0.55 (0.38, 0.81) | **0.002** |
|  | Boys rural | 0.45 (0.27, 0.76) | **0.003** |
|  | Girls cities | reference |  |
|  | Girls towns/suburbs | 1.08 (0.81, 1.44) | 0.619 |
|  | Girls rural | 0.97 (0.68, 1.38) | 0.876 |
| R^2^ = 0.041 | |  |  |

**Table S5. Multiple linear regression analysis for the assessment of hair cortisol concentrations in Flemish adolescent girls**

| **HCC** | **(n )Variable** | **β (95%CI)** | **p-value** |
| --- | --- | --- | --- |
|  | **Age** |  | 0.800 |
|  | (62) [13.5, 14.5] | reference |  |
|  | (142) [14.5, 15.5] | 1.08 (0.86, 1.36) | 0.526 |
|  | (16) > 15.5 | 1.10 (0.72, 1.68) | 0.655 |
|  | **Perceived income adequacy** |  | 0.140 |
|  | (62) Difficult | reference |  |
|  | (70) Rather easy | 0.79 (0.61, 1.02) | 0.069 |
|  | (88) Easy to very easy | 0.81 (0.63, 1.04) | 0.095 |
|  | **Population density** |  | 0.809 |
|  | (141) < 600 inhabitants/km^2^ | reference |  |
|  | (79) ≥ 600 inhabitants/km^2^ | 0.97 (0.79, 1.20) | 0.809 |
|  | **Atopic disease** |  | 0.365 |
|  | (156) No | reference |  |
|  | (64) Yes | 1.11 (0.89, 1.38) | 0.365 |
|  | **Optimal vitality** |  | 0.401 |
|  | (191) No | reference |  |
|  | (29) Yes | 0.88 (0.65, 1.29) | 0.401 |
|  | **Menarcheal status** |  | **0.006** |
|  | (22) Pre-menarche | reference |  |
|  | (198) Post-menarche | 1.59 (1.14, 2.23) | 0.006 |
| R^2^ = 0.035 | |  |  |

**Table S6. Linear regression analyses for the assessment of determinants of hair cortisol concentrations in adolescents, outlying datapoints winsorized**

| **Characteristic** | **Simple linear regression** | | **Linear regression, adjusted for sex and age** | |
| --- | --- | --- | --- | --- |
|  |  |  |  |  |
|  | **Estimate (95% CI)** | ***p*-Value** | **Estimate (95% CI)** | ***p*-Value** |
| **Sociodemographic variables** | | | | |
| Sex |  |  |  |  |
| Boys (191) | reference |  | reference |  |
| Girls (228) | 1.12 (0.97, 1.30) | 0.111 | 1.13 (0.98, 1.30) | 0.098 |
| Age |  | 0.171 |  | 0.154 |
| [13.5 - 14.5] years (113) | reference |  | reference |  |
| [14.5 - 15.5] years (273) | 1.07 (0.91, 1.26) | 0.412 | 1.08 (0.91, 1.27) | 0.379 |
| > 15.5 years (33) | 1.32 (0.99, 1.77) | 0.060 | 1.33 (1.00, 1.78) | 0.054 |
| Country of birth |  | 0.232 |  | 0.306 |
| Belgium (337) | reference |  | reference |  |
| EU (35) | 0.91 (0.70, 1.18) | 0.463 | 0.87 (0.97, 1.13) | 0.310 |
| Outside EU (46) | 1.19 (0.94, 1.50) | 0.145 | 1.13 (0.89, 1.43) | 0.310 |
| Household educational attainment |  | 0.861 |  | 0.879 |
| Primary (24) | reference |  | reference |  |
| Secondary (139) | 1.04 (0.76, 1.43) | 0.803 | 1.08 (0.79, 1.49) | 0.612 |
| Tertiary (249) | 1.00 (0.74, 1.35) | 0.992 | 1.08 (0.79, 1.46) | 0.644 |
| Equivalent income (monthly) |  | 0.381 |  | 0.502 |
| 0 - 1250 Euro (89) | reference |  | reference |  |
| 1251 - 1600 Euro (73) | 1.05 (0.83, 1.31) | 0.698 | 1.05 (0.84, 1.32) | 0.676 |
| 1601 - 2000 Euro (71) | 0.97 (0.77, 1.22) | 0.796 | 0.98 (0.78, 1.23) | 0.842 |
| > 2000 Euro (118) | 0.88 (0.72, 1.07) | 0.203 | 0.90 (0.73, 1.10) | 0.289 |
| Perceived income adequacy |  | 0.092 |  | 0.133 |
| Difficult (119) | reference |  | reference |  |
| Rather easy (133) | 0.88 (0.73, 1.06) | 0.170 | 0.89 (0.74, 1.07) | 0.208 |
| Easy to very easy (160) | 0.82 (0.69, 0.98) | 0.030 | 0.83 (0.70, 1.00) | 0.046 |
| Area deprivation index |  | 0.405 |  | 0.351 |
| 0 - 5.3 % (103) | reference |  | reference |  |
| 5.4 – 9.3 % (105) | 1.08 (0.90, 1.31) | 0.402 | 1.09 (0.90, 1.31) | 0.366 |
| 9.4 – 15.5 % (103) | 1.14 (0.95, 1.38) | 0.169 | 1.15 (0.95, 1.38) | 0.150 |
| >15.5 % (107) | 1.16 (0.96, 1.40) | 0.121 | 1.17 (0.97, 1.41) | 0.096 |
| Urbanicity |  | 0.198 |  | 0.188 |
| Cities (57) | reference |  | reference |  |
| Towns and suburbs (303) | 0.85 (0.69, 1.05) | 0.136 | 0.87 (0.70, 1.07) | 0.191 |
| Rural areas (59) | 0.79 (0.60, 1.03) | 0.082 | 0.78 (0.59, 1.02) | 0.069 |
| Population density |  |  |  |  |
| ≤ 600 /km^2^ (268) | reference |  | reference |  |
| > 600/km^2^ (151) | **1.17 (1.01, 1.35)** | **0.043** | **1.17 (1.00, 1.35)** | **0.045** |
| **Lifestyle variables** | | | | |
| Smoking |  | 0.863 |  | 0.951 |
| Never or once (400) | reference |  | reference |  |
| Less than daily (8) | 1.04 (0.62, 1.76) | 0.877 | 1.03 (0.61, 1.75) | 0.901 |
| Daily (10) | 1.13 (0.71, 1.82) | 0.602 | 1.07 (0.67, 1.73) | 0.767 |
| Alcohol use |  | 0.343 |  | 0.509 |
| Never (263) | reference |  | reference |  |
| Less than monthly (93) | 0.96 (0.81, 1.15) | 0.685 | 0.94 (0.79, 1.13) | 0.530 |
| Monthly or more (61) | 1.15 (0.93, 1.41) | 0.200 | 1.09 (0.88, 1.35) | 0.420 |
| Active sports |  | 0.251 |  | 0.212 |
| Never, occasionally (63) | reference |  | reference |  |
| 1 - 2 times /week (152) | 1.20 (0.97, 1.50) | 0.098 | 1.22 (0.98, 1.52) | 0.080 |
| > 2 times /week (202) | 1.13 (0.91, 1.39) | 0.266 | 1.16 (0.94, 1.44) | 0.164 |
| Hair washing frequency |  | 0.842 |  | 0.793 |
| Daily (67) | reference |  | reference |  |
| ≥ 2 times a week (237) | 1.00 (0.82, 1.22) | 0.993 | 0.97 (0.79, 1.19) | 0.759 |
| < 2 times a week (114) | 1.05 (0.84, 1.32) | 0.680 | 1.02 (0.81, 1.29) | 0.837 |
| Hair treatment |  |  |  |  |
| No (409) | reference |  | reference |  |
| Yes (6) | 0.81 (0.44, 1.49) | 0.503 | 0.72 (0.39, 1.33) | 0.295 |
| **Health characteristics** | | | | |
| Body mass Index |  | 0.221 |  | 0.300 |
| Normal weight (302) | reference |  | reference |  |
| Underweight (34) | 0.91 (0.70, 1.19) | 0.482 | 0.90 (0.69, 1.17) | 0.435 |
| Overweight, obese (83) | 1.15 (0.96, 1.37) | 0.142 | 1.12 (0.93, 1.34) | 0.227 |
| Waist circumference |  | 0.296 |  | 0.286 |
| ≤ 68 cm (101) | reference |  | reference |  |
| [68-72] cm (109) | 1.09 (0.89, 1.34) | 0.385 | 1.10 (0.89, 1.34) | 0.379 |
| [72-78] cm (101) | 1.17 (0.95, 1.44) | 0.139 | 1.18 (0.96, 1.46) | 0.116 |
| >78 cm (106) | 1.20 (0.98, 1.48) | 0.076 | 1.20 (0.98, 1.47) | 0.082 |
| Infections (past year) |  |  |  |  |
| No (162) | reference |  | reference |  |
| Yes (244) | 0.97 (0.83, 1.12) | 0.943 | 0.95 (0.82, 1.11) | 0.524 |
| Atopic disease |  |  |  |  |
| No (281) | reference |  | reference |  |
| Yes (129) | 1.10 (0.96, 1.28) | 0.178 | 1.10 (0.95, 1.27) | 0.184 |
| Corticosteroid medication |  |  |  |  |
| No (409) | reference |  | reference |  |
| Yes (8) | 0.93 (0.58, 1.50) | 0.768 | 0.96 (0.60, 1.55) | 0.870 |
| Menarcheal status (girls) |  |  |  |  |
| Pre-menarche (23) | reference |  | reference |  |
| Post-menarche (205) | **1.61 (1.21, 2.13)** | **0.001** | **1.58 (1.19, 2.11)** | **0.002** |
| Oral contraceptives (girls) |  |  |  |  |
| No (189) | reference |  | reference |  |
| Yes (39) | 1.08 (0.86, 1.36) | 0.525 | 1.04 (0.82, 1.32) | 0.725 |
| SDQ Total difficulties score |  | 0.340 |  | 0.652 |
| Normal (0-15) (325) | reference |  | reference |  |
| Borderline (16-19) (57) | 1.03 (0.83, 1.27) | 0.790 | 1.00 (0.81, 1.23) | 0.987 |
| Abnormal (20-40) (35) | 1.22 (0.94, 1.58) | 0.143 | 1.13 (0.87, 1.48) | 0.362 |
| SDQ Prosocial scale score |  | 0.652 |  | 0.540 |
| Normal (6-10) (381) | reference |  | reference |  |
| Borderline (5) (22) | 1.12 (0.81, 1.55) | 0.476 | 1.14 (0.82, 1.57) | 0.441 |
| Abnormal (0-4) (14) | 0.89 (0.60, 1.33) | 0.578 | 0.86 (0.57, 1.28) | 0.451 |
| Perceived stress |  | 0.681 |  | 0.417 |
| Low (113) | reference |  | reference |  |
| Medium (164) | 1.07 (0.89, 1.27) | 0.480 | 1.05 (0.87, 1.25) | 0.614 |
| High (139) | 1.00 (0.83, 1.20) | 0.983 | 0.93 (0.77, 1.13) | 0.478 |
| Optimal vitality |  |  |  |  |
| No (360) | reference |  | reference |  |
| Yes (56) | **0.75 (0.61, 0.93)** | **0.008** | **0.77 (0.62, 0.94)** | **0.012** |
| Perceived happiness |  |  |  |  |
| Low (34) | reference |  | reference |  |
| High (382) | 0.91 (0.70, 1.18) | 0.486 | 0.98 (0.75, 1.28) | 0.870 |
| Preterm birth (< 37 weeks) |  |  |  |  |
| No (387) | reference |  | reference |  |
| Yes (25) | 1.05 (0.77, 1.42) | 0.774 | 1.08 (0.79, 1.46) | 0.632 |
| Low birth weight (<2.5 kg) |  |  |  |  |
| No (372) | reference |  | reference |  |
| Yes (24) | 0.98 (0.71, 1.34) | 0.881 | 0.93 (0.68, 1.27) | 0.647 |
| Maternal smoking during pregnancy |  |  |  |  |
| No (354) | reference |  | reference |  |
| Yes (58) | 1.06 (0.86, 1.31) | 0.585 | 1.03 (0.84, 1.28) | 0.751 |
| **Meteorology** | | | | |
| 3-month average temperature |  | 0.119 |  | 0.129 |
| <6 °C | reference |  | reference |  |
| 6 – 12 °C | 1.03 (0.89, 1.20) | 0.685 | 1.03 (0.88, 1.20) | 0.713 |
| >12 °C | 1.20 (1.00, 1.43) | 0.047 | 1.19 (1.00, 1.43) | 0.050 |
| 3-month average UV radiation |  | 0.208 |  | 0.142 |
| <300 J/m^2^ | reference |  | reference |  |
| 300 – 1000 J/m^2^ | 1.03 (0.87 (1.22) | 0.716 | 1.02 (0.86, 1.21) | 0.817 |
| > 1000 J/m^2^ | 1.14 (0.98, 1.34) | 0.093 | 1.15 (0.99, 1.35) | 0.071 |
| Season of sampling |  | 0.445 |  | 0.494 |
| Winter | reference |  | reference |  |
| Spring | 1.03 (0.87, 1.21) | 0.745 | 1.03 (0.86, 1.22) | 0.770 |
| Summer | - | - | - | - |
| Fall | 1.13 (0.93,1.37) | 0.218 | 1.12 (0.92, 1.36) | 0.245 |
| **Effect modification by sex, models adjusted for sex and age** | | | | |
| Urbanicity x Sex | | | | **0.014** |
| Population density x Sex | | | | **0.022** |

Estimates (β) are presented with their 95% confidence intervals (95%CI) as the factor change in HCC compared to the HCC of the reference category or the opposite situation for dichotomous variables. Significant associations are marked in bold. ^a^physical activity that causes sweating and/or heavy breathing, ^b^Body Mass Index classes based on age- and sex-specific Belgian growth curves, ^c^doctor-diagnosed asthma and/or atopic dermatitis and/or allergic rhinitis, ^d^cut-off value of study population mean plus one standard deviation ^.^ Abbreviations: EU European Union, SES socioeconomic status, SDQ Strengths and Difficulties Questionnaire.
